# O “perigo da demora” no acesso ao aborto legal no Brasil: a experiência de 111 mulheres

**DOI:** 10.1590/0102-311XPT230925

**Published:** 2026-07-06

**Authors:** Debora Diniz, Ilana Ambrogi

**Affiliations:** 1 Universidade de Brasília, Brasília, Brasil.; 2 Anis: Instituto de Bioética, Brasília, Brasil.

**Keywords:** Aborto, Saúde Reprodutiva, Aborto Legal, Violência Sexual, Abortion, Reproductive Health, Legal Abortion, Sexual Violence, Aborto, Salud Reproductiva, Aborto Legal, Violencia Sexual

## Abstract

O artigo analisa as barreiras enfrentadas por mulheres, adolescentes e meninas no acesso ao aborto legal no Brasil, com base no monitoramento do trabalho do Projeto Vivas, uma organização da sociedade civil de acesso à informação sobre aborto legal, entre fevereiro e novembro de 2025. O estudo faz uma interlocução à decisão do Supremo Tribunal Federal, que negou urgência ao pedido de ampliação da atuação de profissionais da enfermagem em procedimentos de aborto legal. Este é um estudo de recorte temporal-descritivo de barreiras enfrentadas para o acesso ao aborto legal no Brasil, em particular a necessidade de deslocamento. Cento e onze mulheres precisaram deslocar-se de suas cidades para realizar o aborto, percorrendo em média 1.307km, e metade delas mais de 1.000km. A quase totalidade dos casos era de estupro; 67,5% buscaram o Projeto Vivas antes da 12ª semana de gestação, quando o procedimento é de baixa complexidade. O perfil das mulheres indica vulnerabilidade social e econômica: 69,3% com renda de até dois salários mínimos e 49,5% negras. As barreiras mais frequentes foram recusa de atendimento, ausência de médicos e de serviços de referência, e exigências equivocadas. As longas distâncias e o tempo de espera resultam em demora de atendimento e, portanto, podem configurar situações de tratamento cruel, desumano ou degradante, segundo a Organização Mundial da Saúde (OMS). Políticas de saúde focalizadas na descentralização da assistência e na ampliação de profissionais capacitados para o aborto legal na atenção primária, como enfermeiros, estão de acordo com as diretrizes da OMS e merecem ser consideradas para o enfrentamento das barreiras identificadas neste estudo.

## Abstract (en)

This study analyzes the barriers women, adolescents, and girls face in accessing legal abortion in Brazil by monitoring Projeto Vivas (a civil society organization for access to information on legal abortion) from February to November 2025. This study dialogues with the decision of the Brazilian Federal Supreme Court, which denied the urgency to the request toward expanding nurses’ role in legal abortion procedures. This time-descriptive study assessed the barriers for access to legal abortion in Brazil, especially the need for travel. In total, 111 women had to travel from their municipalities for an abortion, averaging 1,307km (half had to travel over 1,000km). Almost all cases stemmed from rape; 67.5% sought the Vivas Project before the 12th week of their pregnancy (during which the procedure entails low complexity). Participants’ profile indicates social and economic vulnerability: 69.3% earned an income of up to two minimum wages and 49.5% were Black. The most frequent barriers included care refusal, lack of physicians and referral services, and mistaken requirements. Long distances and waiting times delay care and can configure cruel, inhuman or degrading treatment according to the World Health Organization (WHO). Health policies to decentralize care and expand professionals trained for legal abortion in primary care (such as nurses) agree with WHO guidelines and deserve consideration to address the barriers identified in this study.

## Abstract (es)

Este artículo analiza las barreras que enfrentan las mujeres, adolescentes y niñas para acceder al aborto legal en Brasil a partir del seguimiento entre febrero y noviembre de 2025 del trabajo del Proyecto Vivas, una organización de la sociedad civil que brinda acceso a información acerca del aborto legal. Este estudio plantea un diálogo sobre la decisión del Tribunal Supremo Federal, que había negado urgencia a la solicitud de ampliar el trabajo de los profesionales de enfermería en los procedimientos de aborto legal. Se trata de un estudio temporal-descriptivo acerca de las barreras de acceso al aborto legal en Brasil, en particular la necesidad de desplazamiento. 111 mujeres tuvieron que viajar desde sus ciudades para abortar, cubriendo un promedio de 1.307km; y la mitad de ellas más de 1.000km. Casi todos los casos fueron casos de violación; el 67,5% buscó el Proyecto Vivas antes de la 12ª semana de embarazo cuando el procedimiento es de baja complejidad. El perfil de las mujeres revela vulnerabilidad social y económica: el 69,3% tenían ingresos de hasta dos salarios mínimos; y 49,5% eran negras. Las barreras más frecuentes fueron el rechazo de la atención, la ausencia de médicos y servicios de referencia y las demandas erróneas. Las largas distancias y el tiempo de espera generan retrasos en la atención y, por lo tanto, pueden constituir situaciones de trato cruel, inhumano o degradante según la Organización Mundial de la Salud (OMS). Las políticas de salud centradas en la descentralización de la atención y en la expansión de los profesionales capacitados en aborto legal en la atención primaria, como las enfermeras, cumplen con las directrices de la OMS y merecen tener en cuenta en el enfrentamiento de las barreras identificadas en este estudio.

## Introdução

O aborto legal no Brasil é permitido quando a pessoa é vítima de estupro, está em risco de vida ou quando a gravidez é de feto anencefálico. Há evidências de que o estado de criminalização cria barreiras desnecessárias e injustas ao aborto legal, como demandas por autorizações judiciais ou registros policiais, deslocamento territorial, longas esperas ou exposições a situações vexatórias e intimidatórias, além de escassez de profissionais de saúde capacitados ou moralmente sensíveis ao atendimento ^1^. 

Em fevereiro de 2025, uma ação judicial sobre ampliação de profissionais autorizados aos cuidados para o aborto legal foi apresentada ao Supremo Tribunal Federal (STF; *Arguição de Descumprimento de Preceito Fundamental 1207* - ADPF 1207) ^2^. A ação foi apresentada por associações nacionais de profissionais da enfermagem e não busca alterar os permissivos legais em vigor, apenas solicita que a assistência em saúde seja também autorizada a profissionais de enfermagem, seja por medicamentos ou por aspiração manual intrauterina (AMIU), procedimentos recomendados para as primeiras 11 e 13 semanas de gestação, respectivamente. Em outubro de 2025, a ação recebeu uma autorização provisória permitindo que profissionais da enfermagem oferecessem assistência ao aborto legal, porém a liminar foi cancelada sem ter efeitos concretos ^3^. O argumento do STF foi de que “não haveria perigo na demora” em decidir, ou seja, de que não haveria urgência para uma liminar ^4^.

A fim de contribuir com evidências para a reflexão provocada por essa ação, instalamos um monitoramento diário das atividades do Projeto Vivas (https://projetovivas.org/), principal grupo da sociedade civil que informa e acompanha deslocamentos territoriais de meninas, adolescentes e mulheres em busca de um aborto legal no Brasil. O Projeto Vivas opera virtualmente com linhas de WhatsApp para atendimento de defensorias, secretarias de saúde, delegacias, unidades de saúde, organizações da sociedade civil e das próprias mulheres. As trabalhadoras do Projeto Vivas são capacitadas para informar sobre a legislação brasileira, as opções disponíveis (como o aborto, a maternidade ou o encaminhamento para adoção), são treinadas na ética do aconselhamento da neutralidade moral e do acolhimento. Em casos de mulheres muito vulneráveis, seja em situação de pobreza ou violência grave, o Projeto Vivas oferece assistência psicológica e jurídica, e suporte financeiro para o deslocamento. 

Esta Comunicação Breve apresenta os dados do fluxo de atendimento do Projeto Vivas de 1º de fevereiro a 1º de novembro de 2025. O monitoramento é coordenado pela Universidade de Brasília e pela Fundação Oswaldo Cruz (FIOCRUZ). É um estudo de métodos mistos, de recorte temporal-descritivo de barreiras enfrentadas para o acesso ao aborto legal no Brasil, em particular a necessidade de deslocamento ^5^. As pesquisadoras acessaram dados não identificáveis das mulheres que buscaram o Projeto Vivas, e o consentimento oral para a pesquisa foi individual por ocasião do atendimento. No universo dos dados, foram atendidas uma menina (mais jovem que 14 anos) e duas adolescentes (mais jovens que 18 anos). Como protocolo do Projeto Vivas, casos de meninas ou adolescentes são encaminhados por defensorias públicas estaduais ou serviços de saúde e seguem o protocolo ético de assentimento próprio, além do consentimento da representante legal adulta. 

Os dados foram organizados por categorias sociodemográficas, marco legal para o aborto, tempo gestacional e barreiras de acesso, em particular, a necessidade de deslocamento territorial. Também foram considerados quesitos como a necessidade de apoio financeiro para o deslocamento, se foi preciso o acompanhamento no deslocamento ou não, se houve acesso à licença médica, e o cálculo de tempo entre a data de primeiro contato ou primeiro exame de ultrassom e a data do atendimento. Optamos por anonimizar as localidades de residência das mulheres e o serviço que realizou o procedimento por razões de confidencialidade, mantendo a distância percorrida. Para o cálculo de distâncias percorridas para o acesso ao aborto legal utilizou-se o Google Maps (https://www.google.com/maps/). A pesquisa foi revisada e aprovada pelo Comitê de Ética em Pesquisa em Ciências Humanas da Universidade de Brasília (CAAE: 49043015.6.0000.5540). 

## Dados

Entre 1º de fevereiro e 1º de novembro de 2025, período de espera do julgamento da ADPF 1207 no STF, o Projeto Vivas atendeu 108 mulheres adultas, duas adolescentes e uma menina que tiveram de se deslocar de suas cidades para acessar o aborto legal, seja com viagens interestaduais ou intermunicipais (total 111). Duas foram atendidas em fevereiro, 19 em março, 13 em abril, 9 em maio, 21 em junho, 22 em julho, 13 em agosto, 11 em setembro e uma em outubro. O perfil das mulheres indica vulnerabilidade social e econômica: 69,3% com renda de até dois salários mínimos e 49,5% negras. Ao menos uma em cada cinco não tinha completado ou alcançado o Ensino Médio. Elas tinham de 12 a 43 anos, com a mediana de idade sendo 28 anos. A maioria, 73 (65,7%), tinha até 30 anos. Pelo menos 18 (16,2%) recebiam algum benefício de políticas sociais, como Bolsa Família ou Auxílio Creche. A maioria delas tem filhos (53, 47,7%) ou são cuidadoras de pessoas dependentes (19, 17,1%); e 39 (35,1%) relataram não ter dependentes (Tabela 1). 

**Tabela 1:** Dados demográficos e sobre a assistência às mulheres, adolescentes e meninas que buscaram o Projeto Vivas entre fevereiro e novembro de 2025 e precisaram de deslocamento.

Variáveis	n	%
Mês		
Fevereiro	2	1,8
Março	19	17,1
Abril	13	11,7
Maio	9	8,1
Junho	21	18,9
Julho	22	19,8
Agosto	13	11,7
Setembro	11	9,9
Outubro	1	0,9
Total	111	100,0
Raça/Cor		
Branca	55	49,5
Indígena	1	0,9
Parda	44	39,6
Preta	11	9,9
Total	111	100,0
Distribuição etária (anos)		
< 18	3	2,7
18-19	5	4,5
20-24	25	22,5
25-29	34	30,6
30-34	26	23,4
35-39	14	12,6
40-44	4	3,6
Total	111	100,0
Causa		
Estupro	109	98,2
Malformação fetal	1	0,9
Risco à vida	1	0,9
Total	111	100,0
Escolaridade		
Ensino Fundamental incompleto	6	5,4
Ensino Fundamental completo	5	4,5
Ensino Médio incompleto	13	11,7
Ensino Médio completo	39	35,1
Superior incompleto	28	25,2
Superior completo	20	18,0
Total	111	100,0
Renda familiar * (salários mínimos)		
Menos que 1	17	15,3
Até 2	60	54,0
Mais que 3	34	30,6
Total	111	100,0
Vínculo de cuidado		
É mãe	53	47,7
É cuidadora **	19	17,1
Não possui dependentes	39	35,1
Total	111	100,0
Beneficiária de políticas sociais		
Sim	18	16,2
Bolsa Família	16	14,4
Auxílio Creche	1	0,9
Seguro desemprego	1	0,9
Não	93	83,8
Total	111	100,0
Tempo gestacional ao contactar o Projeto Vivas [autorreportado] (semanas)		
< 12	75	67,6
12 a 14	11	9,9
15 a 19	9	8,1
20 semanas ou mais	12	10,8
Não sabia	4	3,6
Total	111	100,0
Apoio financeiro		
Não	70	63,1
Sim	40	36,0
Sem informação	1	0,9
Total	111	100,0
Tempo entre o primeiro contato com o Projeto Vivas e o atendimento em serviço (dias)		
Até 7	5	4,5
Entre 8 e 14	20	18,0
Entre 15 e 21	15	13,5
Entre 22 e 28	14	12,6
Entre 29 e 35	11	9,9
Entre 36 e 42	4	3,6
Entre 43 e 49	4	3,6
Entre 50 e 56	1	0,9
Entre 57 e 63	1	0,9
Sem informação	36	32,5
Total	111	100,0
Passou por algum equipamento institucional (delegacia, serviço de aborto legal ou defensoria) antes de contactar o Projeto Vivas		
Não	85	76,6
Sim	26	23,4
Total	111	100,0
	Média de tempo (dias) ^#^	Média de distância (em km) ^##^
Tempo gestacional (semanas) ***		
< 20	19	1.253
≥ 20	33	1.685
Média geral	21	1.307
Mediana		1.070

* Renda familiar de todos os que coabitam no local de residência;

** Cuidado de dependentes que não são filhos;

*** Tempo gestacional autorreportado, exceto para 4 adolescentes ou mulheres que não reportaram, e foi utilizado o tempo gestacional reportado no primeiro ultrassom. Duas das quatro que não reportaram o tempo gestacional no primeiro contato estavam com mais de 20 semanas uma vez que o ultrassom foi realizado;

^#^ Média de tempo em dias entre a data do ultrassom e data de atendimento em serviço (n = 75);

^##^ Média de distância em km de ida e volta do município de residência ao serviço.

A quase totalidade dos casos foi de aborto legal por estupro, havendo apenas um caso por risco de vida e outro por anomalia fetal incompatível com a vida do feto após o parto. Uma em cada quatro já havia passado por serviço de aborto legal, delegacia de polícia ou defensoria pública antes de alcançar o Projeto Vivas (26, 23,4%). Quando uma mulher, adolescente ou menina, chega ao Projeto Vivas é porque já experimentou barreiras para acessar o cuidado, como, por exemplo, de acesso à informação. A peregrinação das mulheres por diferentes instituições de cuidado ou proteção de direitos aconteceu por diferentes razões, sendo a mais comum delas a “recusa de assistência”, “ausência de serviço de referência”, “inexistência de médico na cidade” ou exigências equivocadas como vincular o boletim de ocorrência (B.O.) ao atendimento. As consequências da peregrinação já foram documentadas na literatura em saúde pública, descritas também como barreiras para o acesso, e vão desde impactos à saúde física e mental até o atraso ou a impossibilidade de assistência pelo tempo gestacional ^6^ ^,^ ^7^. 

O perfil da mulher vítima de estupro que busca o aborto legal é semelhante ao encontrado em outros estudos sobre aborto no Brasil ^8^ ^,^ ^9^. Uma característica dos dados que apresentamos é o da vulnerabilidade econômica: uma em cada três mulheres solicitou apoio financeiro, pois não possuía recursos mínimos para o deslocamento entre cidades, seja para passagem de ônibus ou alimentação (40, 36%). Como o atendimento em telessaúde para aborto legal é ainda reduzido no país ^10^, a totalidade das mulheres, adolescentes e a menina foram atendidas em modalidade presencial, ou seja, viajaram para acessar os serviços de aborto. Há várias questões operacionais e morais envolvidas na exigência de deslocamento - uma delas é o cuidado dos filhos ou pessoas dependentes, a ausência no trabalho ou na escola, ou mesmo a exigência de informar sobre a ausência, sendo que muitas vítimas de estupro mantêm sigilo sobre o ocorrido, seja pela situação de violência com o agressor ou sofrimento psíquico ^11^. 

Estudos indicam que as chances de internação são significativamente afetadas pela distância entre a residência da mulher e o local de assistência ao aborto legal ^5^ ^,^ ^12^. Com a mediação do Projeto Vivas, as mulheres tiveram de se deslocar entre 21km e 4.888km somente para ir e retornar do serviço onde realizou o aborto, desconsideradas as peregrinações prévias para exames, delegacia, conselho tutelar, defensoria pública. A escolha do serviço foi dada por disponibilidade de vaga ou equipe para assistência, sendo preferência do Projeto Vivas serviços próximos à localidade da mulher, o que não foi possível para a grande parte dos casos. As mulheres que viajaram menos de 100km eram as que estavam próximas de capitais com os poucos serviços de referência no país. A média de quilometragem percorrida foi de 1.307km, sendo que mais da metade delas (60, 54%) precisou viajar mais de 1.000km. Dentre as que estavam com 20 semanas ou mais de gestação (12 mulheres, 10,8% total), todas viajaram, pelo menos, 800km para acessar o aborto legal. As distâncias são barreiras que implicam tempo sem cuidados para uma necessidade de saúde com tempo próprio de urgência, seja pela gravidez ou pelo impacto na saúde física e mental. Dentre as que estavam com 20 semanas ou mais no dia de atendimento pelo Projeto Vivas, os documentos demonstram que, entre o primeiro ultrassom e o procedimento do aborto, a espera foi de, em média, quatro semanas. 

Se o tempo de deslocamento for hipoteticamente contabilizado como tempo de espera para um procedimento em saúde, uma em cada duas mulheres se manteve como em uma fila de espera ininterrupta por cerca de 12 horas (1.000km). Houve mulheres que esperaram por mais de 60 horas (4.800km). É preciso lembrar que são mulheres sobreviventes de estupro e que a quase totalidade delas deslocou-se sozinha. Algumas jamais haviam viajado desacompanhadas na vida. A ausência de casa, do trabalho ou da escola tem implicações abrangentes para a vida, como, por exemplo, risco de perda do emprego por faltas injustificadas. Nenhuma das mulheres pediu licença médica justificada do trabalho, por razões de privacidade e de foro íntimo sobre a violência sofrida. O sentido da urgência para o acesso ao aborto deve ser compreendido de maneira holística para a vida de cada mulher - risco à saúde física e mental, permanência no emprego ou na escola, estabilidade de relações familiares. 

A oferta de aborto legal em menos de 4% dos municípios brasileiros indica uma importante ausência da assistência, resultando em deslocamentos longos e, consequentemente, na suspensão do cuidado à mulher ou menina vítima de estupro ^1^ ^,^ ^5^ ^,^ ^13^. A maioria delas, 75 (67,6%), contactou o Projeto Vivas em um momento anterior à décima segunda semana de gestação. Isso é, estavam em um período gestacional em que o aborto é procedimento de baixa complexidade, podendo ser autoadministrado por medicamentos, coordenado via telessaúde ou feito de forma ambulatorial em nível de cuidados primários por profissionais da enfermagem, parteiras, médicos generalistas e especialistas ^14^. Encontramos uma correlação que necessita ser mais bem investigada entre avanço gestacional e maiores distâncias percorridas: as mulheres que realizaram o procedimento com mais de vinte semanas tiveram de percorrer maiores distâncias. Há estudos que indicam haver correlação entre acesso restrito ou ausência de assistência próxima à residência da mulher com o risco de avanço do tempo gestacional ^15^ ^,^ ^16^ ^,^ ^17^.

### Indicações preliminares

Os dados preliminares que apresentamos apontam para o impacto da criminalização do aborto para o acesso ao aborto legal por vítimas de estupro. As barreiras ao acesso ao direito ao aborto legal, neste estudo principalmente configuradas em longas distâncias e em tempo de espera, deixam mulheres e meninas em risco evitável de agravos à saúde. Os dados sobre o perfil e a necessidade de deslocamento de 111 mulheres, adolescentes e a menina podem subsidiar o debate público sobre a implementação da assistência ambulatorial, de baixa complexidade, e por profissionais da enfermagem em nível comunitário, demonstrando a urgência do estado de necessidade em curso ^18^. O procedimento do aborto no primeiro trimestre é, de acordo com a Organização Mundial da Saúde, um cuidado em saúde a ser oferecido por vários profissionais, não devendo ser ato privativo de profissionais médicos.

## Data Availability

As fontes de informação utilizadas no estudo estão indicadas no corpo do artigo.
